# Menstrual stigma and the role of culture and religion in the south Asian diaspora: a cross-sectional study of a nationwide cohort

**DOI:** 10.3389/frph.2026.1929160

**Published:** 2026-10-19

**Authors:** Mehnaz Shafquat, Felicia Reid, Gene Urrutia, Ruta Uttarkar, Prema Vyas, Kristen Matteson, Shilpa M. Darivemula

**Affiliations:** 1 School of Medicine, University of North Carolina at Chapel Hill, Chapel Hill, NC, United States; 2 Clinical Affairs Department, Cystic Fibrosis Foundation, Bethesda, MD, United States; 3 Milken School of Public Health, George Washington University, Washington, DC, United States; 4 Division of General Obstetrics and Gynecology, Department of OBGYN, University of North Carolina at Chapel Hill, Chapel Hill, NC, United States; 5 School of Medicine, University of Texas Southwestern Medical Center, Dallas, TX, United States; 6 Division of Graduate Medical Education, Department of Obstetrics and Gynecology, Kaiser, Santa Clara, CA, United States; 7 Division of General Obstetrics and Gynecology, Department of Obstetrics and Gynecology, University of Massachusetts T.H. Chan School of Medicine, UMass Memorial Hospital, Worcester, MA, United States; 8 Division of General Obstetrics, Gynecology, and Midwifery, Department of Obstetrics and Gynecology, University of North Carolina at Chapel Hill, Chapel Hill, NC, United States

**Keywords:** bleeding, cultural barriers, menstrual inequity, menstrual stigma, south Asian diaspora

## Abstract

**Background:**

Menstruation is a physiologic aspect of reproductive health; however, menstrual equity—defined as access to products, supportive environments, and comfort during menstruation—is not universally achieved. Menstrual stigma, often shaped by cultural and religious norms, is a key driver of inequity. While this has been described in global South Asian populations, its influence among South Asians in the United States remains underexplored.

**Objectives:**

To assess the association between cultural and religious influences, menstrual stigma, and menstrual equity among South Asian menstruators in the U.S.

**Methods:**

This cross-sectional study (March–November 2024) recruited South Asian-identifying menstruators via social media. Participants completed a validated 33-item Menstrual Attitudes Questionnaire assessing beliefs, experiences, and menstrual equity indicators. Descriptive statistics and univariate linear regression were used for analysis.

**Results:**

Of 101 respondents, 86% identified as Indian, 53.5% reported menarche at age 11–12, and 59.4% reported regular cycles. Heavy menstrual bleeding was reported by 11.9%. Non-standard menstrual products were used by some participants, including toilet paper (20.6%), paper towels (7.5%), and rags (3.1%). Greater reported cultural or religious influence on menstrual beliefs was associated with denial of any menstrual effect (*p* = 0.001).

**Conclusion:**

Cultural and religious influences were associated with denial of menstrual effects but were not directly associated with overall menstrual stigma. These findings suggest that cultural frameworks may shape menstrual perception in nuanced ways, potentially reinforcing barriers to menstrual health discussion and education. Further research is needed to clarify how cultural and religious norms influence menstrual equity and health-seeking behaviors in South Asian American communities.

## Introduction

Menstruation marks the physiological shedding of the uterine lining through changes in the female reproductive hormonal cycle and serves as a marker of puberty and sexual growth (1–4). Despite being a global phenomenon central to human biological reproduction, many misconceptions and existing cultural biases persist surrounding menstruation (1, 2). Such stigmatizing beliefs have known consequences, including menstruator shame, poor sexual and gynecological healthcare access, unequal access to menstrual supplies, and limited economic and social empowerment for menstruating people (3, 4). These social barriers to menstrual health education and sexual health knowledge contribute to the current epidemic of global menstrual inequity and more broadly, gender inequity (5).

Most of the published literature on cultural biases, stigmas, and misconceptions on menstruation come from low- and middle-income countries, particularly from the South Asian region (6–10). South Asians encompass a wide variety of traditions, customs, religions, and languages representing India, Pakistan, Bangladesh, Nepal, Bhutan, Sri Lanka, and the Maldives. However, despite cultural variations, studies indicate shared regional views of menstruation as a sign of uncleanliness or impurity (6–10). Cultural menstrual biases facilitate the transmission of misinformation about menstruation within families, as adolescent girls often receive their primary menstrual knowledge from mothers and other female relatives (6). This process contributes to the persistence of intergenerational sexual and reproductive health taboos. Many of these cultural menstrual myths have serious and direct health implications on reproductive health (11). Discriminatory cultural practices, such as leaving girls and women out of household activities during their cycles, impact cultural and religious values related to menstruation and continue stigmatization (5, 6). While evidence is limited on the correlation between perceptions, attitudes, beliefs affecting menstrual equity, studies suggest that menstrual shame is associated with worse menstrual outcomes, negative impact on emotional health, menstrual inequity, risk of bacterial infections, and barriers to positive social interactions (5, 12–15).

South Asians are one of the fastest-growing communities in the United States (16). The most recent U.S. Census data indicate that the Asian Indian population experienced a 68% increase between 2000 and 2010 and currently comprises 5.4 million people (16, 17). However, limited research has examined the diverse experiences and perspectives of South Asian menstruators in the United States—a community that may be disproportionately affected by culturally and religiously rooted menstrual biases carried from their countries of origin (18). Given that immigrant families often carry varied home beliefs, intergenerational knowledge, and religio-cultural frameworks upon migration to the United States (19), examining if and how these orientations influence attitudes toward sexual healthcare and menstrual care is critical to achieve menstrual equity in a diverse U.S. population. Such analysis is necessary to identify and address the unique barriers faced by the South Asian diaspora in accessing menstrual products, health education, and clinical care, particularly in light of evidence documenting menstrual stigma and shame across South Asia.

To address this knowledge gap and improve inclusive menstrual equity efforts in the United States, this exploratory national cross-sectional study explores the role of self-reported cultural and religious menstrual influence on the menstrual attitudes, perceptions and experiences of a growing and diverse South Asian community in the United States.

## Materials and methods

### Study design

This study utilized an exploratory cross-sectional design. The multiple-choice survey adapted items from several survey studies in the United States (20–22), published evidence on the Menstrual Attitudes Questionnaire (23), and co-authors’ (RU, PV, SD, MS) diverse lived experiences as South Asian menstruators in the United States. Study recruitment and analysis occurred over a period of 9 months from March to November 2024. Sample size calculations were not performed as responses were not powered for significance for this pilot work.

This study was conducted as a pilot study to inform the design and interview questionnaire for a subsequent funded qualitative study on South Asian menstrual attitudes and experiences. Based on prior published work on menstrual equity in the United States (24), our goal cross-sectional sample size to inform interview questionnaire development was approximately 235 respondents, however, recruitment was truncated to move forward with the established timeline for the subsequent study.

### Ethical approval

Study design, virtual informed consent for all survey participants, and additional study procedures were approved by the University of North Carolina Institutional Review Board (UNC IRB#23-1835). Proceeding to complete the survey was an indication of informed consent.

### Participant eligibility

Individuals were eligible for this study if they identified as (1) South Asian in origin (born in a South Asian country, including Afghanistan, Bhutan, Indian, Pakistan, Nepal, Bangladesh, Sri Lanka, and Maldives or have at least one parent born in any of the aforementioned countries), (2) experiences either regular or irregular menstruation and (3) have a uterus. Additionally, participants must be 18 years or older, primarily English-speaking, and a current resident of the United States. Those who were pregnant at the time of the survey were excluded.

### Participant recruitment and sample derivation

Participants were recruited through a combination of respondent-driven sampling and snowball sampling through social media networks. An online flier with a QR survey code was also distributed through multiple social media channels. Selected social media channels included South-Asian focused U.S. based activist communities on Instagram, Facebook, and Google Groups. The flier was additionally circulated by the American Medical Women's Association (AMWA) after publishing a podcast on the intent of this work (25). Upon accessing the QR code, the participant was presented with the study information sheet to opt in or out of survey completion. Initially, these methods resulted in a seed group of 22 respondents. Respondents were asked to share the survey with at least 5 other eligible people. Anywhere from one to five referrals were enrolled from this original seed group. The largest respondent driven sampling yield which led to an additional forty-five women enrolled was jump started by one participant from the initial seed group who shared the flier on her Whatsapp groups. This dual method of sampling resulted in our sample size of 156 surveys initiated. Any survey with >50% of questions unanswered were removed from the final cohort (Figure 1).

**Figure 1:**
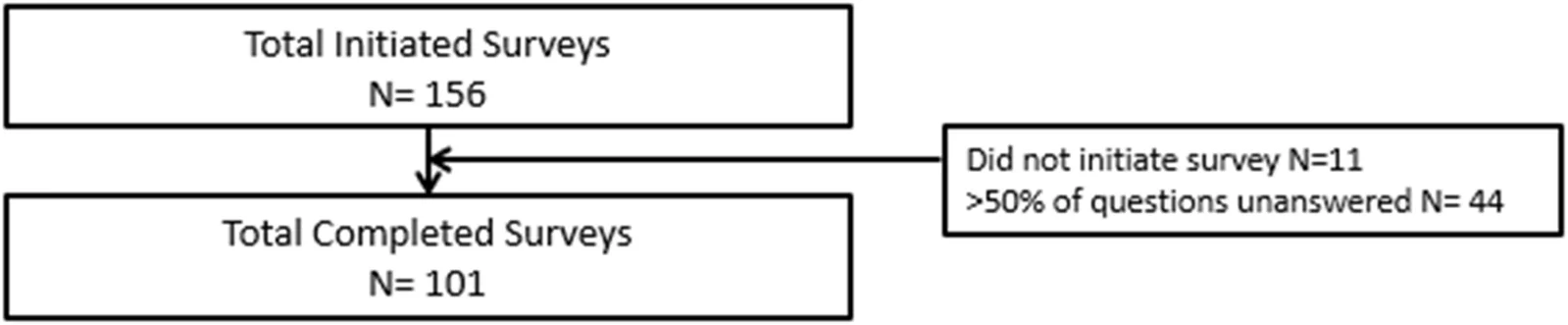
Participant survey enrollment and completion.

### Data analysis

Responses to demographic information were analyzed descriptively, with categorical variables reported as self-identified race and ethnicity, South Asian identity (Indian, Pakistan, Bangladesh, Nepal, Sri Lanka, the Maldives, Bhutan), state of residence, immigration status (‘Born in the US’, ‘Immigrated to the US before age 18’. ‘Immigrated to the US after age 18’, ‘None of the above’), self-described religious practice, highest level of education, and relationship status (‘Not in a relationship at this time’, ‘Not married and in a committed relationship’ ‘Married’, ‘Widowed’, ‘Divorced’, ’Separated’, ‘Prefer not to answer’). Responses to the demographic questions were presented as response frequencies and percentages. Eleven surveys were removed from analysis due to total nonresponse (Figure 1). Surveys with greater than half of the questions left unanswered were removed from final analysis (Figure 1). Any individual missing variable or item level nonresponse was relabeled as a missing variable. Linear regression statistical models were performed on Python 3.13. Paired t-tests were performed using R 4.1.2.

### Primary analysis

Primary analysis was performed to examine the association between level of cultural values’ and level of religious values’ influence on view of menstruation and overall attitudes relating to menstruation. Cultural influence was measured by the response to the question: “How influential are cultural values on your views of menstruation?”. Likewise, religion was measured by the response to the question: “How influential are your religious beliefs on your views of menstruation?”. The Likert-scale responses to both questions were mapped from qualitative to quantitative as: ‘Never Comfortable':1, ‘Rarely comfortable':2, ’Somewhat comfortable':3, ‘Quite Comfortable':4, ‘Extremely comfortable':5.

The Menstrual Attitudes Questionnaire (MAQ) is a 33- item measure of beliefs about menstruation and beliefs regarding general menstruator and personal menstruating experiences (23). Developed in 1970, the MAQ has been validated among adolescents and adults in the United States and globally, including in India, and is widely regarded as the international standard measure for comparing menstrual attitudes (26). Menstrual attitudes were categorized into 5 subscales: 1) Menstruation as a debilitating event, 2) menstruation as a bothersome event, 3) menstruation as a natural event, 4) anticipation and prediction of the onset of menstruation and 5) denial of any effect of menstruation. Each of the 5 categories contained multiple questions, and each question was mapped to a quantitative score where higher agreement with the categorical statement was given a higher score (see Appendix). Subsequently, scores within a category were aggregated as an average score. A small number of respondents left 1 or more questions blank. In such cases, the aggregate mean was calculated after removing the blank response. As a secondary analysis, the total aggregate across all 5 categories was also calculated and modeled. While the MAQ-33 does not specifically measure “stigma”, it includes both positive and negative attitudes, with categories measuring menstruations as “bothersome” and “debilitating” potentially addressing any cultural or religion-driven menstrual stigmatization (27).

Age and race were included as a covariate based on existing literature emphasizing these factors as impacting menstrual experience and attitudes. Note, that respondents were able to select multiple responses for race (e.g., East Asian, South Asian). Five binary covariates were created, one for each of the race choices. Univariate linear regression was utilized to model the effects of the two exposures and six outcomes. All models were fit independently, resulting in twelve final models. Linear regression was selected as the analytical model because both influence and attitude were measured using Likert-scale responses, which, although discrete, can be reasonably approximated as continuous and normally distributed. Sensitivity analyses stratified by race or gender were not conducted due to insufficient sample sizes in all categories except the most common group (“South Asian, Female”). Moreover, age and race were already included as covariates in the regression model, mitigating the need for subgroup-specific analyses.

### Secondary analysis

Based on published evidence of South Asian diaspora indicating avoidance of care due to discomfort with the gynecologist, a secondary analysis was performed to examine whether level of cultural values’ and level of religious values’ influence on view of menstruation affected 1) comfort with gynecologist, 2) comfort with family, or 3) comfort in society. Each of the 3 categories contained multiple questions, and each question was mapped to a quantitative score (see appendix). Age and race were included as covariates and univariate linear regression was utilized to model the effects. All models were fit independently, resulting in six models from two exposures and three outcomes.

### Descriptive analysis

Descriptive responses to questions were not included in either primary or secondary analysis. Each descriptive question was mapped to a quantitative score. To quantify all questions regarding any change from the current state compared to experiences growing up, we performed a paired t-test to determine statistical significance. In addition, participants were also given the opportunity to further elaborate on their scaled views of menstruation in the forms of optional free responses throughout the questionnaire.

## Results

### Demographics

One hundred and fifty-six menstruating people started the survey, of which one hundred and one respondents met completion requirements for inclusion. Participants who completed the survey were between ages of 18–59 years of age, were more likely to have a graduate degree (59%), were married (54%,), immigrated to the United States (63%), and were self-identified followers of Hinduism (68%). Any demographic responses with cell sizes less than 2 were not reported to minimize the risk of deductive disclosure. Table 1 outlines additional demographics.

**Table 1:** Select demographic characteristics of survey respondents (*n* = 101) .

Demographics	*N* **(%)**
Age	
18–29	32 (31.7)
30–39	25 (24.8)
40–49	31 (30.7)
50–59	13 (12.9)
Self-identified Ethnicity/National Heritage	
Bangladesh	7 (7)
India	89 (86)
Pakistan	5 (5)
Relationship Status	
Not in a relationship	34 (33)
In a committed relationship	11 (11)
Married	56 (54)
Highest Level of Education	
High school degree or GED	6 (6)
Some college but no degree	4 (4)
Bachelor's Degree	31 (30)
Graduate Degree	61 (59)
Religion	
Christianity	6 (6)
Islam	10 (10)
Hinduism	71 (68)
Other^a^	11 (11)
No Religion	10 (10)

a Other includes Sikhism, Buddhism, Agnostic, Atheist, and Spiritual grouped together due to *n* < 2 for each individual group.

### Menstrual cycle and hygiene characteristics

Most of the respondents were between the ages of 11–12 years (53.5%) at menarche and reported having regular cycles (59.4%). Approximately one in ten respondents (11.9%) characterized their bleeding as heavy, with some respondents reporting use of non-standard hygiene products, such toilet paper (20.6%), paper towels (7.5%), and rags (3.1%). When asked about menstrual hygiene product knowledge, most respondents were either moderately familiar (34.7%) or very familiar (43.6%) with various options, with sanitary pads serving as the most common product used (53.8%) followed by tampons (16.2%). Table 2 outlines additional menstrual supply use and bleeding characteristics.

**Table 2:** Frequency of responses regarding menstrual characteristics and hygiene experience (*N* = 101).

Questions on menstrual characteristics		*N* (%)
Age at first period		
9–10		8 (7.9%)
11–12		54 (53.5%)
13–14		34 (33.7%)
15–16		5 (5.0%)
Cycle regularity		
Regular		60 (59.4%)
A mix of both		26 (25.7%)
Irregular		15 (14.9%)
Bleeding characterization		
Light		10 (9.9%)
Moderate		52 (51.5%)
A mix of the above		27 (26.7%)
Heavy		12 (11.9%)
Product familiarity		
Not familiar at all		0 (0.0%)
Slightly familiar		8 (7.9%)
Moderately familiar		35 (34.7%)
Very familiar		44 (43.6%)
Extremely familiar		14 (13.9%)
Products currently used		
Sanitary pads		98 (53.8%)
Tampons		38 (20.9%)
Period panties		18 (9.9%)
Cloth/rags/towels		12 (6.6%)
Menstrual cups		11 (6.0%)
Other		5 (2.7%)
Products ever used		
Pads/sanitary napkins		100 (43.9%)
Toilet paper		47 (20.6%)
Tampons		37 (16.2%)
Paper towels		17 (7.5%)
Cups		13 (5.7%)
Rags		7 (3.1%)
Other		4 (1.8%)
Diapers		3 (1.3%)

### Univariate linear regression modeling

The primary analysis, which controlled for age and self-reported race and ethnicity, suggested that respondents whose views of menstruation were more strongly influenced by either cultural values (*p* = 0.001) or religious values (*p* = 0.001) were more likely to deny any effect of menstruation (Table 3).

**Table 3:** Univariate linear regression analysis between culture and religion on MAQ-33 aggregate responses (*n* = 101).

Questions on culture and religion	How influential are cultural values on your views of menstruation effect (95% CI)	How influential are your religious beliefs on your views of menstruation? effect (95% CI)
Menstruation as a debilitating event (negative)	0.07 (−0.05, 0.19)	0.09 (−0.02, 0.21)
Menstruation as a bothersome event (negative)	−0.02 (−0.16, 0.11)	0.04 (−0.09, 0.17)
Menstruation as a natural event (positive)	0.17 (−0.03, 0.37)	0.11 (−0.09, 0.3)
Anticipation and prediction of the onset of menstruation (positive)	0.05 (−0.1, 0.2)	0.02 (−0.12, 0.16)
Denial of any effect of menstruation (positive)	0.29 (0.12, 0.46)	0.27 (0.11, 0.44)
Overall Positive View	−0.04 (−0.1, 0.02)	−0.07 (−0.13, −0.01)

Secondary analysis suggested evidence that respondents who reported being more influenced by culture were slightly more likely to feel comfortable with their gynecologist. An increase of 1 point on the Likert scale for influence of cultural values on views of menstruation was associated with a 0.13 point increase in average Likert scale response to questions associated with “Comfort with gynecologist” [95% CI (0.03, 0.23)].

## Discussion

In this analysis of South Asian identifying menstruators in the U.S. diaspora, there was no association between self-identified religious and cultural menstrual influence and overall menstrual attitudes as measured by the Menstrual Attitude Questionnaire (Table 3). Interestingly, in subscale analysis, those who did identify that culture or religion influenced their view of menstruation were more likely to deny any effect of menstruation on their lives (Table 4). Respondents who identified culture influencing menstrual views were slightly more comfortable with their gynecologist (Table 4). While effect size was near negligible given small sample size, these results imply that negative perceptions of menstruation and menstrual stigma are not directly dependent on self-rated cultural and religious influence on menstrual views in the South Asian diaspora community.

**Table 4:** Univariate linear regression modeling assessing the influence of self-reported culture and religious beliefs with self-reported comfort (*n* = 101).

Questions on self-reported comfort	How influential are cultural values on your views of menstruation: effect (95% CI)	How influential are your religious beliefs on your views of menstruation? effect (95% CI)
Comfort with gynecologist	0.13 (0.03, 0.23)	0.08 (−0.02, 0.18)
Comfort with family	−0.15 (−0.36, 0.05)	−0.17 (−0.37, 0.03)
Comfort with Society	−0.17 (−0.38, 0.05)	−0.17 (−0.38, 0.03)

Growing research on menstrual attitudes in South Asia—a region with documented beliefs on menstrual pollution and menses-associated social and religious restrictions (27, 28)—has highlighted the dual effects of cultural and religious affinity with menstrual experiences. Crawford et al. conducted a qualitative study of menstrual stigma in Nepalese women and the sources driving meaning-making surrounding cultural and historical evidence of religious menstrual restrictions and stigmatization (27). Surprisingly, Crawford noted that most of their Hindu Nepalese sample adhered to the idea that menstruation is a natural process, rationalizing any aspect of menses-associated isolation or sense of uncleanliness as “rituals as part of a longstanding cultural tradition”, “discrimination against women”, or as a “celebration of womanhood”. Crawford further describes the difference between “enacted stigma”—or “overt discrimination due to socially unacceptable actions like cooking”—and “felt stigma”—or internalized sense of shame for any social violation in this community; coping strategies such as “picking and choosing which ones [restrictions] to observe” used by the Nepalese participants to reduce enacted stigma was driven mainly by avoiding experiencing felt stigma (27). Interestingly, familial culture drove much of the experience—if any—of enacted stigma, with more modern and urban families experiencing less. A 2025 study of urban Bangladeshi women found that regardless of job status or education, most menstruators believed their family members were generally supportive of their needs during their cycles (29). This general positive community response towards menstruation was also noted in a 2022 study of Indian students from South India, where participants overall expressed a positive attitude towards menstruation (30). Overall, our findings align with this recent research and indicate that previously highlighted South Asian religious and cultural menstrual restrictions and stigma 1) depend on family, specifically maternal figures, reaction and enforcement and 2) may be welcomed as markers of religious adherence and of womanhood.

One of the costs of welcoming menstruation and concentrating transmission of menstrual knowledge within communities and families is missing signs of abnormal menstruation. Several menstrual abnormalities were noted in this study and were not highlighted as respondent concerns. One in ten respondents confirmed abnormal heavy bleeding (11.9%); abnormal heavy bleeding has been associated with increased risk of depression and anemia—two clinical outcomes that are also common in the South Asian community (31, 32). Nearly half of the respondents notably reported early menarche, with their first period coming between ages 9–10 (7.8%) and between 11 and 12 (53.5%)—an unexpected result of this survey. Early menarche is defined as getting your first period before age 12 (33). Menarche is a complex phenomenon determined by genetic and environmental factors. However, early menarche has been on the rise, with increased body mass indices, high sugar consumption, and early exposure to endocrine disrupting chemical as associated drivers (33). Early menarche is also associated in a 15%–20% increased risk of breast cancer (34), obesity (35), adverse cardiac events (36), and diabetes (37) in adulthood due to the increased exposure to cyclic estrogen. For South Asians, this is particularly concerning. South Asian immigrants have significantly higher risks for cardiovascular disease and for insulin resistance—even when controlled for BMI—compared to Caucasian and African immigrants and even compared to native South Asians (16, 38). Only 14.3% of respondents indicated extreme familiarity with menstrual products, with several respondents using diapers, rags, and towels to manage their cycles in the United States. A secondary analysis of South Asian American women in the MASALA study noted that nearly a third of women reported premature menopause with significant burdens of hot flashes for over two decades without awareness that early menopause and prolonged vasomotor symptoms are a concern (39). The coexistence of common menstrual abnormalities, limited knowledge of and access to menstrual products, and the normalization of irregular menstrual experiences within family and cultural networks may lead to missed opportunities for early detection and intervention in a population already at elevated risk for adverse health outcomes and menstrual inequities. Examining how cultural and religious norms restrict open dialogue can further illuminate the ways menstrual stigma is experienced among South Asians in the United States.

South Asians in the diaspora have documented barriers to prenatal and gynecological care (39, 40). There are no studies examining the physician-patient relationship through the lens of South Asian identifying patients or physicians in the United States, however, studies from England and Canada identify existence of stereotyping, racism, and bias bilaterally (17). Although this study did not find a strong association between self-identified cultural or religious beliefs and comfort with gynecologic care, it remains plausible that deeply held beliefs could influence the decision to seek such care. The specific content and educational practices within clinical settings, however, are not well understood. Gynecologists should address the intersection of early and abnormal menstruation with the cultural context of South Asian patients to enhance both patient comfort and long-term reproductive health outcomes.

### Limitations and strengths

Most participants identified India as their cultural home (86%) with a majority identifying as Hindu (68%). Almost one third of the cohort had graduate degrees (59%) and over half (54%) were married. Given this small sample size, national and religio-cultural skew, and high level of education—a known protective factor against felt menstrual stigma—these findings may not be generalizable to a large South Asian diaspora population. The 2000 Census data notes that up to 29.5% of the South Asian diaspora live below 125% of the poverty level (17), and so, it is possible that the online method yielded participants of higher socioeconomic status. Additionally, data was not divided by immigration status, so determining the role of acculturation—a known health risk factor—on menstrual felt stigma is not possible. The length of the survey, with MAQ-33 and additional familial and knowledge questions, could have been a deterrent to completing the survey in its entirety. This, along with the lack of financial incentive, could have contributed to the discrepancy between the number of menstruators who started the survey compared to the number who met completion requirements. The survey format supported recall bias.

While these limitations are present, our pilot study exhibited several strengths. First, this study served as a national needs assessment on South Asian menstrual experiences and led to an evidence-based interview guide used in the larger parent study to address some of the identified gaps in menstrual stigma and equity. The online method of data collection also allowed for a diverse sample across all ages of the reproductive lifespan. Use of scales, such as MAQ-33, confirmed outcome alignment with previously published work and identified gaps in menstrual awareness for the South Asian immigrant community. Finally, the study revealed a prevalence for abnormal menstrual experiences worthy of additional clinical and research follow up for South Asians in the United States.

### Future recommendations for research

Further research needs to be done to address South Asian immigrant menstrual needs, divided by level of acculturation and immigration status (recently immigrated vs. those born and raised abroad) to create person-centered counseling frameworks devoid of cultural stereotyping. Efforts to develop a patient-driven, validated scale assessing South Asian specific needs and experiences regarding menstruation, menarche, stigma, and clinical experiences should be pursued, including broad qualitative work and subsequent diverse validation follow up. Evidence from two studies in Nepal (41) and Bangladesh (28) on urban, educated menstruators noted persistence of menstrual stigmas despite financial or educational status; this persistence indicates the cultural influence on menstrual health. Future studies involving qualitative methods may be necessary to elucidate this cultural paradoxical relationship.

Emerging research among South Asian migrant communities suggests that migration and increased exposure to Western social contexts do not necessarily eliminate culturally embedded menstrual beliefs and practices (42). In a 2025 qualitative study of twenty-two Nepali women of mainly high caste and Hindu origin living in the United States, participants described relinquishing menstrual restrictions that interfered with everyday social life while selectively retaining religious practices because of their religious identity, respect for tradition, and sense of community (42). Research among Asian-Indian immigrant women in the United States similarly demonstrates that bicultural integration—between “American” and “Indian” backgrounds— are associated with differences in reproductive and gender-related attitudes, suggesting that cultural beliefs may evolve differently depending on the degree and form of acculturation (43). There are no other primary cohort based studies evaluating the role of migration to the United States on menstrual practices or stigma for a larger, diverse South Asian cohort, indicating a gap in need of further research endeavors. And so, the existing literature does not provide sufficient evidence to directly determine whether menstrual stigma differs between South Asian individuals who immigrated to the United States and those who were born and raised in the United States. Our study was not powered for this analysis and future studies should include comparative efforts to determine this possible causal relationship between migration, acculturation, and menstrual stigma. Finally, education for gynecologists on providing culturally sensitive care should be included to enhance equity.

## Conclusion

South Asians represent a substantial segment of the United States population but remain underrepresented in gynecological health research. This gap limits understanding of menstrual health experiences in a community with distinct cultural, familial, and migratory contexts that shape both health behaviors and clinical engagement.

While menstrual stigma has often been conceptualized through a predominantly Western framework emphasizing silence, shame, and restriction, our findings and existing literature suggest a more nuanced reality in South Asian communities. Menstruation may also be embedded within cultural constructions of womanhood, familial support systems, and religious or traditional practices that shape meaning-making around menses. These dynamics underscore the need for further research that moves beyond binary interpretations of stigma to better understand menstrual management, beliefs, and lived experiences within this diaspora.

Importantly, this study also highlights a concerning burden of unrecognized menstrual abnormalities, including heavy menstrual bleeding and early menarche, alongside limited awareness and access to appropriate menstrual health resources. These findings suggest that clinically significant symptoms may be normalized within cultural and familial frameworks, resulting in missed opportunities for early identification and intervention. Given the elevated cardiometabolic and reproductive health risks already faced by South Asians, improved attention to menstrual health and its intersectional health impacts in this population is urgently needed across both research and clinical practice.

## Data Availability

The raw data supporting the conclusions of this article will be made available by the authors, without undue reservation.
